# Effects of a Music-Based Rhythmic Auditory Stimulation on Gait and Balance in Subacute Stroke

**DOI:** 10.3390/ijerph18042032

**Published:** 2021-02-19

**Authors:** Samira Gonzalez-Hoelling, Carme Bertran-Noguer, Gloria Reig-Garcia, Rosa Suñer-Soler

**Affiliations:** 1 Neurorehabilitation Department, Hospital Sociosanitari Mutuam Girona, 17007 Girona, Spain; samifisioterapia@gmail.com; 2 Health and Health Care Research Group, University of Girona, 17003 Girona, Spain; carme.bertran@udg.edu; 3 Department of Nursing, Faculty of Nursing, University of Girona, 17003 Girona, Spain; gloria.reig@udg.edu

**Keywords:** stroke, rhythmical auditory stimulation, physical therapy, musical therapy, rehabilitation

## Abstract

Gait and balance impairments are common after stroke. This study aimed to evaluate the effect of a music-based rhythmic auditory stimulation (RAS) in combination with conventional physiotherapy on gait parameters and walking ability in subacute stroke. This single-blind, historical controlled trial, included 55 patients who had suffered a stroke within the three weeks prior to enrolment. Patients from 2018 (*n* = 27) were assigned as the historical control group whereas 2019 patients (*n* = 28) received music-based RAS three times a week. Both groups received 11 h of conventional physiotherapy per week during hospitalization. Primary outcomes were gait and balance parameters (Tinetti test and Timed Up&Go test) and walking ability (Functional Ambulation Category scale). Secondary outcomes were trunk control, assistive devices, functional independence (Functional Independence Measure, Barthel index), and stroke severity and disability (modified Rankin scale, National Institutes of Health Stroke Scale). Results: No between-group differences were identified for gait and balance parameters nor for secondary outcomes. Significant between-group differences were observed in the Functional Ambulation Category: the intervention group (Δmean ± SD; 3.43 ± 1.17) showed greater improvement (*p* = 0.002) than the control group (Δmean ± SD; 2.48 ± 1.09). Compared with conventional physiotherapy alone, our results suggest that the walking ability of subacute stroke patients might be improved with music-based RAS combined with conventional physiotherapy, but this treatment is not more effective than conventional physiotherapy in obtaining gait and balance gains.

## 1. Introduction

Stroke is the main cause of disability in adults and the third cause of death in developed countries. It can affect motor function, language, cognition, and perceptual-sensory processing. Hemiplegia and hemiparesis are frequent and stroke reduces the quality of life in those who survive [1].

Music therapy has been found to provide a series of health benefits, including improvements in physiological responses, pain tolerance, motivation to perform physical activity, anxiety levels, attention and memory, and in better social interaction and communication [2,3]. A close relationship has been found between the neural activity of the auditory and premotor cortex during rhythm processing, ratifying that rhythm perception is based on auditory and motor system interactions [4]. Since rhythm is processed in some of the same ways as timing, and timing is essential for many perceptual and motor functions, other music components are needed to process music-based rhythm across multiple brain areas, such as those related to pitch and timbre perception [4].

Rhythmic stimulation improves corporal performance, inducing body movements and stimulating muscle action, which can be observed in some dances [5,6,7]. Rhythmic auditory stimulation (RAS) is a neurological music therapy technique to improve motor control in rehabilitation and therapy by using the physiological effects of the rhythmic motor cueing [7]. An intervention with rhythmic auditory stimulation can be beneficial to improve gait parameters in people in the acute phase as well as in the chronic phase after stroke by increasing gait speed, improving step length of the affected side and cadence, and improving static balance. Quality of life is also enhanced according to the Stroke Specific Quality of Life Scale [7,8,9,10]. A multimodal rhythm and music-based rehabilitation programme may contribute to positive experiences for many individuals after stroke in terms of motor, cognitive, as well as emotional enhancements [11]. Using RAS in people with hemiplegia has been shown to benefit time-space and kinesthetic aspects, correcting gait pattern; improving hip adduction, knee flexion, dorsal and plantar flexion; stimulating bilateral step symmetry; and producing a softer transition between step phases [12,13,14]. While the use of a metronome has been shown to improve stride length, the use of music-based rhythmic auditory stimulation produces more gains in velocity and cadence [10]. Most trials have investigated rhythmic auditory stimulation on gait parameters, such as gait speed and cadence, in subacute and chronic phase after stroke. [10,15] Very little attention has been paid to the role of music-based RAS on walking ability in a subacute phase within the three weeks after stroke onset [15]. After stroke, not only gait parameters but also walking ability has been shown to have an impact on perceived participation after stroke [16]. Walking ability is considered a significant marker for global health ratings and a predictor of health outcomes [17]. Rehabilitation start time also has an important impact on functional outcomes after stroke and must be considered [18].

The present study aimed to evaluate the effect of a music-based rhythmic auditory stimulation in combination with conventional physiotherapy on gait parameters and walking ability in people with stroke in a subacute phase, within the 21 days after stroke onset, and compare it to conventional physiotherapy alone. We hypothesized that people in both groups would improve in gait parameters, walking ability, fall risk, and functional independence, and that participants in the music-based rhythmic auditory stimulation group would produce more gains in gait parameters and walking ability.

## 2. Materials and Methods

### 2.1. Study Design and Participants

This study was an evaluation-blinded, quasi-experimental trial with a historical control group. A convenience sample of 55 people meeting the criteria were enrolled from the multidisciplinary intensive rehabilitation unit for subacute stroke between 2018 and 2019. This research was approved by the Bioethics Committee of the *Unió Catalana d’Hospitals*, registered in *ClinicalTrials.gov* (NCT03974490) and all participants were informed of the study purpose and procedures before signing an informed consent form.

People with the following criteria were included: diagnosis of a first-time stroke (either ischemic or hemorrhagic), or without sequel of a previous stroke, within the preceding three weeks; age > 18 years; previously an independent walker and Barthel score > 85; hemiparesis with gait disturbance after the stroke; Rankin score 3–4, Tinetti score < 23. People with the following criteria were excluded: independent walkers, aphasia that impeded communication, moderate to severe cognitive disorder (Mini-Mental State Examination score < 24), affectation of the posterior cerebral artery territory, previous musculoskeletal or neurological disease, and people who did not wish to participate. The historical control group were people with the same criteria who had a stroke during 2018, when music-based RAS had yet to be introduced as a technique of rehabilitation at the hospital being studied.

### 2.2. Intervention

The intervention group received music-based rhythmic auditory stimulation for 90 min, three times per week, two hours of conventional physiotherapy from Monday to Friday, and one hour of physiotherapy on Saturdays. Participants began the intervention at hospital admission and did sessions until discharge. The number of sessions depended on the days of stay.

The music-based RAS intervention consisted of 15 min of general body warming following the rhythm with a metronome, a main part of the session with 60 min of music-based RAS exercises, and closure with 15 min of relaxation exercises after which each of the participants gave their impression of how the session had gone. The music-based RAS exercises on Mondays and Fridays were with the Ronnie Gardiner Method^®^, which is based on neuroplasticity principles, motor learning and postural control [11,19]. On Wednesday, treatment was based on walking training with music overlaid with a metronome according to the clinical protocol of M.H. Thaut [10]: anterior walking, lateral walking, military march, tandem walking, posterior walking, walking on one’s toes, and then on one’s heels, through progressive variations and increments of rhythm speed (from 50 bpm to 110 bpm). The music chosen was from a variety of past and present musical genres, with a marked pulse, 1/4 or 6/8 rhythm, and variation of beats per minute. Music-based rhythmic auditory stimulation was carried out by a licensed physical and music therapist in the rehabilitation room. (For more information about the intervention, see Appendix A).

The control group did two hours of conventional physiotherapy from Monday to Friday and one hour on Saturday to improve gait and balance. Conventional physiotherapy consisted of therapeutic exercise and walking training in a parallel walking bar or with assistive devices. Therapeutic exercise was based on proprioceptive neuromuscular facilitation, trunk dissociation, motor control and strengthening exercises. Patients with severe hemiplegia and sensorimotor impairments practiced sitting and standing balance and sit-to-stand in the parallel walking bar. As their physical function improved, they progressed to dynamic standing balance and gait training with assistive devices.

The parameters of interest were measured before and after the whole treatment. All evaluations were made by a licensed physiotherapist, except for the modified Rankin scale and National Institutes of Health Stroke Scale, which were assessed by a neurologist. The evaluators did not know if the patient had had a music-based RAS.

### 2.3. Outcome Assessment

The primary outcome measures were standing balance, gait if they could walk, and fall risk assessed using the Tinetti test, the Timed Up&Go test and the gait speed. Walking ability was measured with the Functional Ambulation Category scale.

The Tinetti test evaluates dynamic and static balance and walking patterns. There are 9 balance score categories and 10 gait score categories, scores range from 0 to 28, with higher scores representing better gait and balance and lower fall risk [20]. The Timed Up&Go test measures the time a person needs to stand up, walk 3 meters, turn around, go back to a chair, and sit down. If the time is >20 s, a fall risk is assumed [21]. Gait speed was calculated by timing the seconds needed to walk 10 meters. The Functional Ambulation Category scale categorizes six levels (0 to 5) of gait assistance [22]. We have recoded and dichotomized the variable, defining the patients with an FAC score ≤ 2 as non-walkers and those with an FAC score ≥ 3 as walkers [23].

Secondary outcome measures included trunk control, assistive devices, the Functional Independence Measure, and the Barthel Index. Trunk control was scored as 0 if the patient had no trunk control while sitting; 1 if the patient could keep sitting with external help; and 2 if the patient could keep sitting without losing balance [20]. Assistive devices were categorized in four levels: 0 no assistive device needed, 1 for cane or crutch use, 2 for walker use, and 3 for wheelchair. Functional Independence Measure (FIM) is scored in 18 categories (from 0 to 126) and each item is rated on a 7-point scale. The focus is on motor and cognitive function independence with higher scores [24]. Barthel Index (scored from 0 to 100) is a reliable index for measuring activities of daily living, with higher scores indicating greater independence [25]. The National Institutes of Health Stroke Scale (NIHSS) was evaluated by a neurologist at acute hospital admissions (Dr. Josep Trueta University Hospital), at the study baseline at the subacute and rehabilitation hospital (Hospital Sociosanitari Mutuam Girona), and three months after stroke onset at outpatients at the Dr. Josep Trueta Hospital. This scale measures stroke severity with 15 impairment items, with the sum of the items giving a total score ranging from 0 to 42; the higher the score, the more severe the stroke [26]. The degree of disability or dependence in daily activities is measured with the modified Rankin scale, which runs from 0 to 6 [27]. We recodified the categories in: (1) for no symptoms, no significant disability and slight disability, (2) for moderate disability, (3) for moderately severe disability and severe disability, and (4) for death. This outcome was also evaluated by a neurologist at baseline and discharge from the subacute rehabilitation hospital.

### 2.4. Statistical Analysis

Data were analyzed using SPSS Statistics version 17.9 (SPSS Inc., Chicago, IL, USA). Participant characteristics in each group were examined using non-parametric statistics, Mann–Whitney U tests for continuous variables and Chi-square tests for nominal variables. Mean differences in group outcomes at discharge versus at baseline were compared using Mann–Whitney U tests. We performed a multiple linear regression to study the variables closely associated to the Functional Ambulation Category outcome. In all tests, significance was set at *p* < 0.05.

## 3. Results

**Figure 1:**
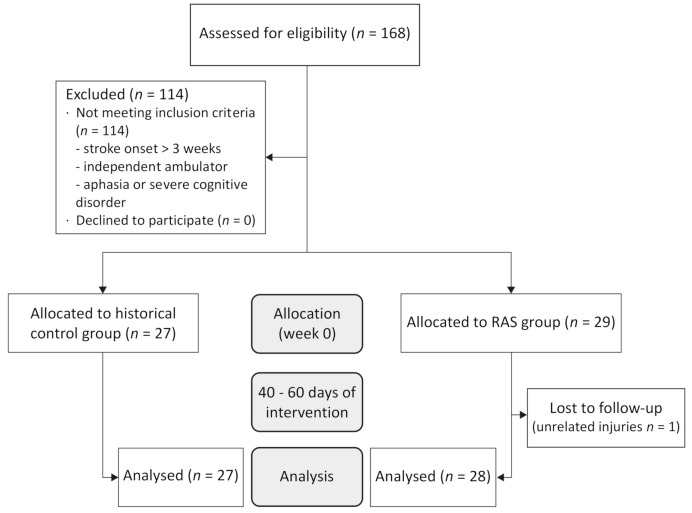
Flow diagram of participants.

In total, 168 people with stroke were screened for eligibility between January 2018 and December 2019, of which 114 were excluded in line with the exclusion criteria. From January to December 2018, 27 people were analyzed and included in the historical control group (hereafter, the control group). from April (when music-based rhythmic auditory stimulation started being used in the hospital) to December 2019, we recruited 29 participants. Among the participants who were lost to follow-up, one participant in the music-based RAS group was diagnosed with pneumonia, which was not considered to be related to the intervention with music-based rhythmic auditory stimulation. During the study, there were no adverse events or safety problems related to the music-based rhythmic auditory stimulation. A flow diagram of participants is presented in Figure 1.

The final analysis included 53 participants: 28 in the music-based RAS group and 27 in the control group. The control group age ranged between 43 and 77, of which 19 participants (70.4%) were male, whereas the age range in the music-based RAS group was between 25 and 83, of which 16 (57.7%) were male. The days after the onset when patients were enrolled ranged from 5 to 21 days in the control group and from 4 to 21 days in the music-based RAS group. The days of stay in the control group ranged from 7 to 97 days and in the music-based RAS group from 16 to 88. The characteristics of the participants for each group are presented in Table 1. Sex, age, stroke etiology, side of hemiparesis, affected area, risk factors, NIHSS at baseline, days from onset and days of stay in the subacute rehabilitation hospital did not differ significantly between the music-based RAS and the control group. The music-based RAS group did between 3 and 34 sessions of music-based rhythmic auditory stimulation, depending on the days of stay. Only one subject did 3 sessions, because of a voluntary discharge after 10 days of stay. If this subject is excluded, an average of 14.22 ± 7.98 (mean ± SD) sessions is obtained. No adverse events were reported during the study.

At baseline, there were no significant differences between groups in the Tinetti score, the Timed Up and Go test, gait speed and the Functional Ambulation Category Table 2. For secondary outcomes such as trunk control, assistive device, Functional Independence Measure, Barthel Index and stroke severity no significant differences were found (*p* > 0.05). The Mann–Whitney U test showed significant differences for the modified Rankin scale, between the two groups, at baseline (*p* = 0.000) and at patient discharge (*p* = 0.001).

**Figure 2:**
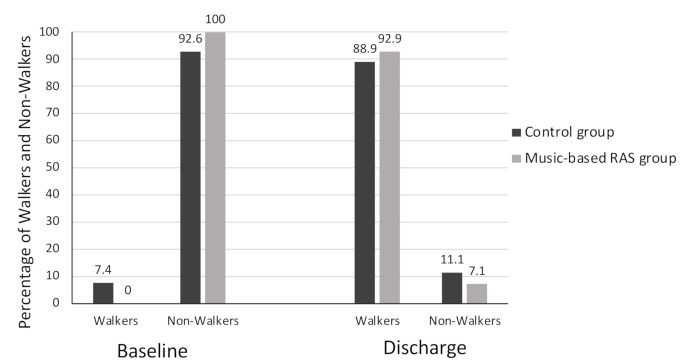
Functional Ambulation Category recodified score by groups at baseline and discharge. Notes: The figure shows the distribution of people (percentage) in the recodified categories of the Functional Ambulation Category from the music-based rhythmic auditory stimulation group and the control group at baseline and discharge. No differences are found (*p* value > 0.05) by the Chi-square test between groups at baseline or discharge.

The patients from both groups had improved significantly by discharge. No relevant differences between the music-based RAS group and the control group were observed for the primary outcomes at patient discharge. The Tinetti minimum score at baseline was 0 for both groups and a maximum of 22 in the control group and 21 in the music-based RAS group; after intervention the minimum was 9 for the control group, 1 for the music-based RAS group, and a maximum of 28 for both groups. The Timed Up & Go improved more than 4 s and reduced fall risk in both groups. Twenty-one (77.8%) of the people in the music-based RAS group and 18 (64.3%) in the control group could not walk at all at baseline, thus gait speed was 0 m/s. Gait speed at the patients’ discharge ranged between 0 m/s and 1.07 m/s in the control group and from 0 m/s to 1.30 m/s in the music-based RAS group. Twenty-five subjects (92.6%) in the control group and 28 (100%) in the music-based RAS group could not walk at baseline as defined by the Functional Ambulation Category. At discharge, 8 (29.6%) participants in the control group could walk with supervision, 9 (33.3%) could walk indoors, and 7 (25.9%) could walk outdoors independently; in the music-based RAS group 6 (21.4%) people could walk with supervision, 15 (53.5%) could walk indoors and 5 (17.9%), could walk outdoors independently Figure 2.

**Figure 3:**
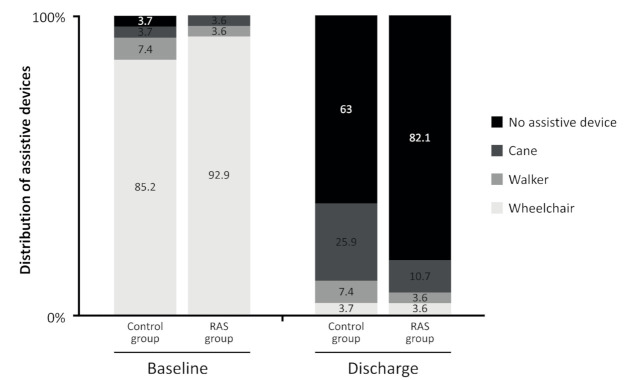
Assistive devices by groups at baseline and discharge. Note: The figure shows the distribution (as percentages) of assistive devices used by the participants in each group at baseline and at discharge. No differences are found (*p* value > 0.05) by Chi-square test between groups at baseline or discharge.

Interestingly, even though no difference was found at discharge between the two groups in walking ability, mean differences between discharge and baseline showed a significant (*p* = 0.002) improvement in the music-based RAS group with the Functional Ambulation Category Table 3 compared to the control group. The second set of outcomes revealed that trunk control improved in both groups so that 98% of people could sit on their own. Assistive devices changed as shown in Figure 3: at baseline 23 (85.2%) participants in the control group and 26 (92.9%) participants in the music-based RAS group needed a wheelchair. At discharge, 17 (63%) in the control group and 23 (82.1%) in the music-based RAS group could walk without any assistive device, but between-groups difference was not significant (*p* > 0.05).

The Functional Independence Measure and the Barthel Index clearly improved in the two groups. At baseline, 23 participants (85.2%) from the control group were in category 3 (moderately severe disability) on the modified Rankin scale, and in the music-based RAS group 15 (53.6%) participants were in category 3 and 12 (42.9%) in category 2 (moderate disability). At patient discharge, people from the control group were in category 1 (*n* = 17; 63%) and 2 (*n* = 9; 33.3%), compared with the music-based RAS group where 27 participants (96.4%) improved to category 1—no significant or slight disability (Chi-square test; *p* = 0.004). As shown in Table 3, improvement was measured by calculating differences in the mean score between discharge and baseline. The Mann–Whitney U test was used to compare outcome improvements between the music-based RAS group and the control group, and no differences were revealed for secondary outcomes between the two groups.

In the multiple linear regression that was performed to analyze factors associated to Functional Ambulation Category outcome (age, sex, affected area and side, stroke severity at baseline and intervention group), only stroke severity measured at baseline with the NIHSS showed a significant correlation at patient discharge (β = −0.136; *p* = 0.005).

## 4. Discussions

This study suggested that music-based rhythmic auditory stimulation in combination with conventional physiotherapy was more beneficial for people with subacute stroke than conventional physiotherapy alone. When contrasted with the control group, it is seen that people who received music-based rhythmic auditory stimulation showed significant improvements in functional ambulation and walking ability. Our research was unable to demonstrate that the music-based RAS group improved more in gait parameters, fall risk, trunk control, functional independence, and independence in activities of daily living, when compared with the control group.

Despite potential differences in the mechanism of action for music-based rhythmic auditory stimulation and conventional physiotherapy [15,28,29], the goal of music-based RAS alone and in combination remained the same: to improve gait and balance after stroke [8,14,28,30]. A possible explanation might be related to the lack of precision in the Tinetti test to measure gait parameters and the quality of walking in the aftermath of stroke [14,31]. Consistently with previous literature, we found that rhythmic auditory stimulation in combination with conventional physiotherapy was not effective in improving balance [8,32]. Our research gave similar results for the two groups with regards to gait and fall risk, using the Timed Up&Go test, unlike other studies [14,28,31,33]. Great improvement in both groups may be due to the intensive multidisciplinary rehabilitation in the stroke unit. Limited gains in the music-based RAS group may be related to some patients’ inability to tolerate the extra therapy sessions [34]. This discrepancy could be attributed to the clinical phase of the participants. Most studies have been undertaken in the chronic phase whereas our research was with people with no more than 21 days after stroke onset [10,12]. It should be taken into account that the control group also attended an intensive rehabilitation unit, performing 11 h of physiotherapy and 7 h of occupational therapy per week, compared with control groups of other studies that only received an average of 3–5 h/week. This undoubtedly has value for rehabilitation but possibly reduces the comparative effect with the intervention group [10,15,34]. Another possible cause could be that we could not evaluate the Timed Up&Go and Tinetti scores in those participants who were unable to walk at baseline (more than 80% in each group). Little was found in the literature about the effect of music-based rhythmic auditory stimulation on walking ability stated with the Functional Ambulation Category scale in acute and subacute stroke patients. Yoo and Kim (2016) found no differences between the stage of stroke subgroups with regards to the effects of rhythmic auditory stimulation on gait parameters, but larger effect sizes on walking velocity and cadence were observed in acute stroke compared to chronic stroke [10]. Peurala et al. (2009) showed that walking training improved gait functions irrespective of the method used [35]. Our most obvious finding from the analysis was that the music-based RAS group were more independent in walking at discharge (if we added together indoors and outdoors walkers) than the control group. We corroborated the results of Kim et al. (2012) that gait abilities in people with subacute stroke improve with rhythmic auditory stimulation [33]. Outcomes such as severity of the affected side (hemiparesis vs. hemiplegia) or muscle strength were not considered in our research, although previous studies have shown the correlation of muscle strength as a predictor of walking ability [16,36].

In the second set of research outcomes, the need for assistive devices was in line with the walking ability of our main aim. More than the 80% of the music-based RAS group went home without any assistive device, compared with the 63% in the control group. In reviewing the literature, no data was found on the association between music-based rhythmic auditory stimulation and the use of assistive devices. The reason for a similar improvement in trunk control in both groups is not clear but it may be related to the focus of the treatment being on standing and walking stability, and the sensitivity of the scale used [37]. The Functional Independence Measure and the Barthel Index of functional independence were not found in the literature to have been evaluated in relation to the effect of music-based rhythmic auditory stimulation on gait and balance, in people with subacute stroke. It seems possible that our results are due to interference with the arm function. These scales measure independence in daily living activities, where arm function is needed [34]. The Modified Rankin scale and the NIHSS have been used in stroke literature to describe the demographic characteristics of study participants, but we have not found previous research about disability improvement after a music-based rhythmic auditory stimulation [11,31]. Despite this, we have found in our study that a music-based rhythmic auditory stimulation was able to improve disability from moderate or severe at baseline to slight or no disability at discharge. With respect to intervention sessions, the music-based RAS group had four hours per week more of intervention than the control group. Our results do not coincide with Kwakkel et al. (2004), who found that increasing exercise therapy had a small favorable effect on activities of daily living [34]. The cost-effectiveness for difference in hours of intervention in our study is an important issue which we will consider in future research.

This trial had several limitations. The size of the sample has not allowed us to identify more differences between the two groups. As they were groups of 27 and 28 people, there is a risk of a bias when the value of a variable of one or two people differs significantly from the median [38,39]. We used a design with a historical, non-parallel, control group, and no randomization was performed, which limits the robustness of the study. This was necessary as the Bioethics Committee, to ensure the principle of beneficence, required us to perform a music-based rhythmic auditory stimulation with every patient who could benefit from the therapy and so did not allow us to establish a control group with conventional physiotherapy alone. The findings of our study showed few differences in improvement between the two groups, which should help us to obtain approval from the Ethics Committee for future randomized controlled trials.

A second limitation is the cognitive impairment inclusion criteria assessed with the Mini-Mental State Examination. A minimum score of 24 was insufficient, given that this scale has modest qualities in screening for low and mild cognitive disturbances in people with stroke. More cognitive functions could be affected after stroke in people with scores between 27 and 30 (normal cognitive function) than those functions that are evaluated with this measure [40].

A third limitation is that the number of music-based rhythmic auditory stimulation sessions in the music-based RAS group was not determined by the researcher. The days of stay and intervention sessions depended on the achievement of therapeutic objectives as assessed by the rehabilitation hospital. Even so, an average of 15 sessions was undertaken by the patients, similar to other trials [12].

Finally, the intervention was not individualized, which we consider as both a limitation and a strength. Participants in a walking phase were together with non-walkers, which made it difficult to find the right level of challenge. However, social relationships and motivation were established between the participants in the music-based RAS group, consistent with some literature [8].

Strengths of this trial include the collection of walking ability outcomes, such as functional ambulation or the use of assistive devices enabling music-based rhythmic auditory stimulation to be applied to walking independence. The mixture of methods used in the music-based RAS group: exercises with metronome, walking exercises, the Ronnie Gardiner Method, and the variability of music, may have helped to encompass different dimensions of the music-based therapy [7,8,10]. Music is found to be connected to emotional responses through an associative learning process; therefore, physical response and executive function can be influenced and enhanced more than by just a marked rhythm from a metronome [7]. Therapy satisfaction has recently been shown by Wang et al. (2021) to be higher when music-based stimulation is used [41]. In order to maintain the metronome rhythm, gait speed is adapted subconsciously but Forner-Cornero et al. (2019) found that the relation between the foot contact and the sound cue showed a mean error which increased when frequencies changed [42]. When frequencies changes from baseline, the foot contact tend to be before the sound cue. Forner-Cornero suggested that two processes might be involved in rhythm entrainment, one a slow-adapting, supraspinal oscillator, which predicts the foot contact, and a second fast process related to sensory inputs, which adapts peripheral sensory input (foot contact) and supraspinal sensory input (auditory rhythm) [42]. Studies into supraspinal feedback errors with a music-based rhythmic auditory stimulation should be planned in future research. As suggested by Van Criekinge et al. in 2019, our research was with people in a subacute stroke phase rather than a chronic phase and used actual pieces of music besides a metronome [15]. Future research needs to investigate the cost-effectiveness and the persistence of treatment effects over time and at people’s homes with larger cohorts. The recording of variables such as the FAC or the use of assistive devices has probably provided a more functional and qualitative value to the evaluation of the ambulation, unlike the usual parameters such as the cadence or the length of the step, which do not reflect the degree of autonomy of the patient or their walking ability [43]. In future publications we will report the effects of a music-based rhythmic auditory stimulation on the severity of stroke impairments at 12 months.

## 5. Conclusions

This study is one of the first to evaluate the effect of a music-based rhythmic auditory stimulation in combination with conventional physiotherapy, compared to conventional physiotherapy alone, on walking ability in people with subacute stroke. Participants in both groups improved in balance, gait, fall risk, trunk control, and functional independence. However, music-based rhythmic auditory stimulation was not found to produce gains in gait parameters. This study shows that music-based rhythmic auditory stimulation adds value to functional ambulation and walking ability. More studies with blinded and randomized control trial methods are needed.

## Figures and Tables

**Table 1:** Participant characteristics by group.

	Control Group (*n* = 27)	Music-Based RAS Group (*n* = 28)	*p*-Value
**Age (years)**	62.2 ± 8.9	65.7 ± 12.7	0.246
**Sex (*n*)**			0.335
Male	19 (70.4)	16 (57.7)	
Female	8 (29.5)	12 (42.3)	
**Stroke etiology (*n)***			0.922
Hemorrhage	9 (33.3)	9 (34.6)	
Infarction	18 (66.7)	17 (65.4)	
**Side of hemiparesis (*n*)**	–	–	0.491
Right	20 (74.1)	17 (65.4)	
Left	7 (25.9)	9 (34.6)	
**Affected area (*n*)**	–	–	0.453
Basal ganglia	6 (22.2)	6 (23.1)	
MCA	7 (25.9)	6 (23.1)	
Vertebrobasilar	3 (11.1)	7 (26.9)	
Lacunar	5 (18.5)	4 (15.4)	
MCA + ACA	1 (3.7)	1 (3.8)	
Thalamus	1 (3.7)	1 (3.8)	
Cerebellar	4 (14.8)	1 (3.8)	
**Risk factors (*n*)**	–	–	–
Arterial hypertension	17 (63.0)	19 (67.9)	0.703
Diabetes Mellitus type 2	11 (40.7)	11 (39.3)	0.912
Dyslipidemia	9 (33.3)	12 (42.9)	0.467
Heart disease	9 (33.3)	7 (25)	0.496
Obesity	5 (18.5)	10 (35.7)	0.152
**Toxic habits**	–	–	–
Tobacco	4 (14.8)	5 (17.9)	0.348
Alcohol	4 (14.8)	1 (3.6)	
Mental health disorders	3 (11.1)	4 (14.3)	0.724
**NIHSS (*n*)**	–	–	0.583
Acute hospital admissions	–	–	
Josep Trueta Hospital	9.1 ± 5.3	8.9 ± 6.4	
Median (IQR)	8 (5–13)	8 (3.3–11)	
**Days from onset (days)**			0.600
	10.07 ± 4.27	10.46 ± 4.09	
Median (IQR)	9 (7–11)	10 (7–14)	
**Days of stay (days)**	–	–	0.293
Subacute and rehabilitation	–	–	
Mutuam Hospital	52.3 ± 24.9	45.7 ± 20.6	
Median (IQR)	48 (36–73)	43.5 (26–62.2)	

Notes: Values are presented as mean ± SD or number (percentage). MCA: Middle cerebral artery; ACA: Anterior cerebral artery; NIHSS = National Institutes of Health Stroke Scale; (IQR) = Interquartile range. Chi-square test for sex, stroke etiology, side of hemiparesis, affected area and risk factors. Mann–Whitney *U* test for age, NIHSS, days from onset and days of stay.

**Table 2:** Outcome scores at baseline and discharge.

Outcome	Baseline			Discharge		
	Control Group (*n* = 27)	Music-Based RAS Group (*n* = 28)	*p*-Value	Control Group (*n* = 27)	Music-Based RAS Group (*n* = 28)	*p*-Value
**Tinetti score**	9.8 ± 7.5	8.3 ± 6.8	0.389	24.1 ± 4.3	23.1 ± 5.8	0.593
(max score = 28)	9 (3–16)	8 (1–14)		26 (21–27)	24.5 (22–27)	
**Timed Up and Go**	16.5 ± 4.8	20.5 ± 11.9	0.79	12.6 ± 10.8	14.0 ± 6.1	0.058
(seconds)	16.4 (12.7–20.4)	17.1 (3.6–24.3)		10.4 (6.6–13.4)	12.4 (10.1–16.0)	
**Gait Speed**	0.1 ± 0.2	0.1 ± 0.2	0.314	0.5 ± 0.2	0.6 ± 0.3	0.314
(meters per second)	0.0 (0)	0.0 (0)		0.5 (0.3–0.6)	0.6 (0.4–0.9)	
**FAC**	1.2 ± 0.6	0.4 ± 0.7	0.142	3.7 ± 1.2	3.8 ± 1.1	0.696
(max score = 6)	1 (1–1)	0 (0–0.7)		4 (3–5)	4 (3–4)	
**FIM**	87.9 ± 17.2	85.5 ± 19.6	0.99	119 ± 9.2	120.0 ± 6.9	0.638
(max score =126)	86 (78–97)	88 (72.7–98)		122 (120–124)	121 (120–124)	
**NIHSS ^#^**	5.1 ± 3.0	5.6 ± 3.5	0.622	1.6 ± 1.8	0.7 ± 2.2	0.036
(max score = 42)	4 (3–7)	5 (3–8)		1 (0–2.5)	2.5 (1–3)	
**Barthel index**	42.2 ± 14.7	48.1 ± 21.7	0.254	92.6 ± 10.3	91.1 ± 13.7	0.646
(max score = 100)	45 (30–55)	45 (35–63.7)		95 (90–100)	92.5 (90–100)	

Notes: Values are presented as mean ± standard deviation, or median (interquartile range). FAC = Functional Ambulation Category; FIM = Functional Independence Measure score; NIHSS = National Institutes of Health Stroke Scale; max score = maximum possible score of test or scale. # = National Institutes of Health Stroke Scale at baseline in subacute and rehabilitation hospital and at three months after stroke onset in outpatient consultation at the Dr. Josep Trueta University Hospital of Girona. No differences are found (*p* value > 0.05) by the Chi-square test between groups at baseline or discharge.

**Table 3:** Between-group mean differences in change, discharge versus baseline.

Outcome	Control Group (*n* = 27)	Intervention Group (*n* = 28)	*p*-Value
Tinetti score	14.30 ± 6.71	14.71 ± 7.37	0.840
	15 (8–20)	14 (8.5–20.75)	
Gait speed	0.36 ± 0.19	0.53 ± 0.26	0.621
FAC score	2.48 ± 1.09	3.43 ± 1.17	0.002 *
	3 (2–3)	3.5 (3–4)	
FIM score	31.07 ± 17.58	34.57 ± 16.70	0.544
	33 (22–43)	35 (23–44.5)	
Barthel Index	50.37 ± 13.65	44.29 ± 20.98	0.326
	50 (45–60)	47.5 (31.25–55)	

Values are presented as mean difference ± standard deviation, or median (interquartile range). FAC = Functional Ambulation Category; FIM = Functional Independence Measure score; (IQR) = interquartile range. * *p* < 0.05, by the Mann–Whitney U test for mean differences (change between baseline and discharge).

## Data Availability

Dataset could be found on http://dx.doi.org/10.17632/dsngw3zsnz.1 (accessed on 17 February 2021).

## Appendix A

Research was conducted in a stroke unit from the subacute and rehabilitation hospital Mutuam in Girona, Spain. Patients did conventional physiotherapy in the rehabilitation room; music-based RAS intervention on Mondays and Fridays was in the function room and music-based RAS intervention on Wednesday was in the rehabilitation room.

*1. Intervention*

Music-based rhythmic auditory stimulation for 90 min consisting of

-General body warming (15 min):

Following the rhythm with a metronome, between 40 and 60 bpm. People who could keep standing balance did it standing without assistive devices, those with severe hemiplegia did it sitting down until they could stand by themselves:

*cervical flexion-extension and rotation

*shoulders rotation

*trunk rotation

*pelvic movements

*shoulders and pelvic dissociation

*knees flexion-extension

*ankle flexion-extension

-Main part (60 min)

Mondays and Fridays were with the multi-task exercises from Ronnie Gardiner Method^®^. The therapist projects symbols as hands and feet on the wall, red for left and blue for right side of the body, each symbol means a movement and is related with a sound. The patients follow the symbols beat by beat, increasing the tempo during the session. Ronnie Gardiner Method^®^ has many symbols, but in study sessions we only used 5 symbols. Patients had a red sticker on the left hand, and a blue sticker on the right hand in order to facilitate the perception of the two body sides.

Music pieces used:

∘ Twinkle twinkle little star (68 bpm)
∘ One love-Bob Marley (76 bpm)
∘ No woman no cry-Bob Marley (78 bpm)
∘ Lemon Tree-Fool’s Garden (143 bpm)
∘ Tea for two cha cha (instrumental)-Tommy Dorsey Orchestra (167 bpm)

On Wednesday, walking training with music and a metronome overlaid, according to the clinical application guide of M.H. Thaut [7]:

anterior walking, lateral walking, military march, tandem walking, posterior walking, walking with toes and then with heels, through progressive variations and increments of rhythm speed (from 50 bpm to 110 bpm), accentuation of every beat and on beat depending on the exercise. 8 min each exercise and 2 min rest between them. The music chosen was from a variety of past and present musical genres, with marked pulse, 1/4 or 6/8 rhythm, and variation of beats per minute. People who could not walk, participated by weight shifting standing in stationary position in parallel bars or holding on to other assistive device.

Music pieces used:

∘ The lion sleeps tonight-The Tokens (68 bpm/121 bpm)
∘ Colonel Hathi’s March, The Jungle Book-J. Pat Omalley (96 bpm)
∘ Someone you loved-Lewis Capaldi (110)
∘ Yellow submarine-The Beatles (111 bpm)
∘ Radetzky march from Johann Strauss-performed by Peter Guth and the Royal Philharmonic Orchestra (111 bpm)
∘ Ayo Technology-Milow (128 bpm)
∘ Old Town Road-Lil Nas X (136 bpm)
∘ Je veux-Zaz (155)
∘ Lost on you-LP (174 bpm)
∘ Qué vendrá-Zaz (176 bpm)

- Closure (15 min):

Relaxation exercises with deep breathing, body stretching while sitting and a round of impressions to know about the mood and motivation with the music-based therapy.

Music pieces used:

∘ River flows in you-Yiruma
∘ Canon D minor-Pachelbel
∘ Enya-Only time
∘ Intermezzo from Cavalleria Rusticana-Pietro Mascagni

*2. Setting and material*

The interventions on Mondays and Fridays were in the rehabilitation room, 20 × 25 m, one side with chairs to make the warm-up and closure and one side without obstacles to perform the main walking part.

Wednesday: Over-ground walking on a 10 × 2 m floor with lateral bars.

Metronome: Metronome & Tuner TGI 99B

LED projector: Manufacturer VPRAWLS, 1080p full HD, 1200 lumens, contrast ratio 1000:1, diagonal visible screen 50 cm × 127 cm.

Speaker: EasyAcc LX-839, 3W, 20 Hz–90 Hz, with Bluetooth and Micro SD.

We used a Micro SD with music pieces downloaded from https://www.youtube.com platform (accessed on 17 February 2021).
